# Benefits and harms of citrate locking solutions for hemodialysis catheters: a systematic review and meta-analysis

**DOI:** 10.1186/s40697-015-0040-2

**Published:** 2015-04-02

**Authors:** Alexa Grudzinski, Arnav Agarwal, Neera Bhatnagar, Gihad Nesrallah

**Affiliations:** Department of Basic Medical Sciences, Faculty of Science, Western University, London, ON Canada; Faculty of Medicine, University of Toronto, Toronto, ON Canada; Department of Clinical Epidemiology and Biostatistics, McMaster University, Hamilton, ON Canada; Lawson Health Research Institute, Western University, 375 South Street, London, ON Canada

## Abstract

**Background:**

Citrate has theoretical advantages over heparin for locking hemodialysis central venous catheters (CVCs), but the comparative effectiveness of these agents is not clear.

**Objectives:**

1) To compare the benefits and harms of citrate versus heparin locking solutions among patients undergoing hemodialysis through CVCs; 2) to appraise methodological quality of the supporting evidence.

**Data sources:**

CENTRAL, MEDLINE, EMBASE, CINAHL, ISI Web of Science, and nephrology conference abstracts.

**Study eligibility, participants, and interventions:**

We included randomized, parallel arm clinical trials that enrolled adult patients (>18 years) receiving chronic hemodialysis through CVCs using a citrate locking solution. We excluded studies in which citrate was combined with other agents, such as antibiotics.

**Appraisal and synthesis methods:**

We used the GRADE approach to systematic reviews and quality appraisal. Two reviewers performed data extraction independently and in duplicate. We pooled count data using generic inverse variance with random-effects models, and used fixed-effect models when only two studies were available for pooling. Subgroups included low (≤5%) vs. higher (≥30%) citrate.

**Results:**

We screened 600 citations. Forty-one proceeded to full-text screen; 5 met inclusion criteria. Studies included between 19 and 291 participants (Median N = 61) followed for a total of 174.6 catheter-years; 2 were multi-centred trials. Three studies assessed all-cause mortality; the pooled relative risk for death was 0.71 (95% CI = 0.42-1.24; p = 0.21; I^2^ = 0%). The rate ratio for bacteremic episodes was 0.54 (95% CI = 0.23-1.29; p = 0.16; I^2^ = 65%) while the rate ratio for bleeding was 0.48 (95% CI = 0.3-0.75; p = 0.001;I I^2^ = 5%). Rates of catheter exchange/replacement, all-cause hospitalization and in-situ thrombolysis were not significantly different between groups in any of the pooled analyses. Risk of bias within pooled studies was low.

**Limitations:**

Outcome definitions varied across studies. Imprecision due to small sample sizes and low event rates reduce our overall confidence in the pooled effect estimates.

**Implications:**

Benefits and harms of citrate vs. heparin locking solutions remain unclear; larger studies and standardization of outcome measurement and reporting are warranted.

**Trial registration:**

Protocol Registration Number: CRD42013004781

**Electronic supplementary material:**

The online version of this article (doi:10.1186/s40697-015-0040-2) contains supplementary material, which is available to authorized users.

## Abrégé

**Contexte:**

En théorie, il appert que les solutions de citrate présentent certains avantages, comparativement aux solutions héparinées, lorsqu’il s’agit du verrouillage des cathéters veineux centraux (CVC) utilisés en hémodialyse. Cependant, l’efficacité relative de ces deux agents reste incertaine.

**Objectifs de l’étude:**

1) Comparer les avantages et les désavantages des solutions de verrouillage de citrate, d’une part et d’héparine, d’autre part, chez les patients subissant des traitements d’hémodialyse par CVC, et 2) évaluer la qualité méthodologique des données probantes.

**Sources d’information:**

Les bases de données CENTRAL, MEDLINE, EMBASE, CINAHL, ISI Web of Science, et des abrégés de communications à des conférences en néphrologie.

**Admissibilité de l’étude, choix des participants et interventions:**

Nous avons tenu compte des essais cliniques à répartition aléatoire à deux groupes, effectués sur des adultes (18 ans et plus) sous traitements réguliers d’hémodialyse par CVC, et dont le verrouillage utilisait une solution de citrate. Les essais dont la solution de verrouillage combinait le citrate à d’autres agents, comme des antibiotiques, ont été exclus.

**Méthodes d’évaluation de la qualité de la preuve et de synthèse:**

Nous avons utilisé la méthode GRADE pour analyser la qualité des données. Une extraction des données a été réalisée de manière indépendante par chacun des deux évaluateurs. Nous avons regroupé les données de dénombrement en utilisant un modèle générique de type « inverse variance with random-effect » et avons utilisé un modèle de type « fixed-effect » lorsque nous n’avions que deux études à notre disposition. Les sous-groupes ont été divisés selon la concentration de citrate dans la solution de verrouillage : basse (≤5%), ou élevée (≥30%).

**Résultats:**

Nous avons passé 600 titres en revue. 41 d’entre eux menaient au texte plein écran; 5 rencontraient les critères d’admissibilité. Le nombre de participants pour chaque étude se situait en 19 et 291 (médiane N = 61), pour un total de 174,6 années-cathéter; 2 des études étaient des essais cliniques multicentres. Trois études avaient rendu compte de toutes les causes de mortalité; la mesure combinée du risque relatif de mortalité était de 0,71 (95% IC = 0,42-1,24; p = 0,21; I^2^ = 0%). Le rapport de taux des bactériémies était de 0,54 (95% IC = 0,23-1,29; p = 0,16; I^2^ = 65%), tandis que le rapport de taux des hémorragies était de 0,48 (95% IC = 0,3-0,75; p = 0,001; I^2^ = 5%). La variation dans la fréquence de remplacement/changement du cathéter, d’hospitalisation (toutes raisons confondues) ou de thrombolyse *in situ* n’était pas significative entre les différents groupes et ce, dans toutes les analyses combinées. Le risque de biais à l’intérieur des études regroupées était faible.

**Limites de l’étude:**

La définition des résultats variait selon l’étude. Notre confiance générale dans les estimations regroupées est réduite puisqu’il existe certaines imprécisions résultant de petits échantillons et le taux d’événements est faible.

**Implications:**

Les avantages et désavantages de l’utilisation du citrate, comparativement à l’héparine, dans les solutions de verrouillage restent incertains. Il serait justifié de procéder à des études plus vastes ainsi qu’à une uniformisation de la mesure des résultats et des rapports.

## What was known before

Prior reviews have suggested that citrate-containing antimicrobial locking solutions reduce the risk of bacteremia. However, the quality of the supporting evidence has not been extensively reviewed.

## What this adds

In this review, we focus on outcomes with citrate alone versus heparin-based locking solutions for hemodialysis catheters, and apply the Grading of Recommendations Assessment, Development and Evaluation (GRADE) approach to systematic reviews and evidence quality appraisal. We identify a low overall quality of evidence supporting a lower risk of bleeding, but no difference in infection, hospitalization, or patency-related outcomes with citrate versus heparin.

## Introduction

Despite guidelines, policies, and initiatives promoting arterio-venous fistulae, prevalent central venous catheter (CVC) rates persistently range between 10-50% internationally, with upward trends in many high-income countries, including Canada [1,2]. Catheter-related bacteremia remains a common and potentially catastrophic hemodialysis complication, resulting in significant morbidity and mortality, as well as hospitalization and resource use. Moreover, thrombotic or patency-related complications reduce dialysis adequacy, are costly, and result in treatment disruptions and inconvenience for patients. While unfractionated heparin has been widely used as a locking solution, it is associated with a number of complications, including inadvertent systemic administration with coagulopathy and bleeding [3], heparin induced thrombocytopenia [4], and allergic reactions [5].

Citrate-based locking solutions are a promising alternative to unfractionated heparin, with a number of small clinical trials recently evaluating its efficacy and safety. Moreover, the unit cost for citrate has recently fallen to below that of heparin in Canada and other jurisdictions, making citrate a potentially cost-effective alternative. Our aims were: 1) to conduct a systematic review and meta-analysis to better understand the benefits and harms of using citrate versus heparin as a CVC locking solution, and 2) to appraise the quality of the supporting body of evidence.

## Methods

### Overview

Our review adhered to a pre-specified protocol, registered with the PROSPERO register of systematic reviews (registration number: CRD42013004781) [6]. We used the Grading of Recommendations Assessment, Development and Evaluation (GRADE) approach to systematic reviews and methodological quality appraisal [7-10]. This manuscript was prepared according to the PRISMA Guidelines [11].

### Study eligibility

#### Types of studies

We included randomized clinical trials and systematic reviews published as full-text articles in peer-reviewed journals, as well as abstracts presented at major nephrology conferences. We excluded observational studies, opinion pieces, narrative reviews, and any other articles not containing original data.

#### Types of participants

We included studies that enrolled adult patients (>18 years) with end-stage renal disease undergoing chronic hemodialysis through CVC’s using a citrate locking solution (intervention) and heparin-based locking solutions (control). Given major potential differences in disease severity and treatment settings, we excluded studies of inpatient or acute kidney injury populations, or those admitted to intensive care units, or receiving continuous renal replacement therapies. We also excluded studies in which patients received hemofiltration or hemodiafiltration. We did not place any restrictions on catheter types (tunneled vs. temporary), incident vs. prevalent patients, or incident vs. prevalent catheters.

#### Types of interventions

Experimental interventions consisted of citrate-based catheter locking solutions – both low (4%) and higher (15-50%) concentrations. The control intervention included heparin or other non-citrate based locking solutions.

#### Types of outcomes

Critically important outcomes included patient survival (all-cause mortality), bacteremia rates, and all-cause hospitalization rates. Important but not critical outcomes included access-related hospitalization, catheter replacement/exchange events, bleeding, and local/in situ thrombolysis (tPA, urokinase, or other), as measured by number of thrombolytic administration episodes. We used outcome definitions as they were described in the included studies.

### Information sources

We searched The Cochrane Central Register of Controlled Trials (CENTRAL) [12], MEDLINE, EMBASE, CINAHL and ISI Web of Science from inception to June 29, 2013.

### Search

All search strategies were developed in collaboration with a Health Information Specialist (NB), and included the following headings and text words: population – dialysis, hemodialysis; intervention – citrate, citric acid, sodium citrate; and comparator – heparin, other locking solutions terms identified during pilot searches. A sample search strategy is in the web Additional file 1. We did not restrict the search strategy based on outcome, language or date of publication. Our primary search was supplemented with hand-searches of narrative and systematic review bibliographies. We also searched conference abstracts from the Canadian Society of Nephrology and the American Society of Nephrology annual meetings spanning 2011–2013.

### Study selection

We downloaded all identified reports into a reference manager (Endnote X6 for Macintosh Thomson-Reuters Inc., San Francisco, CA, USA). Two authors (AA and AG) independently assessed all titles and abstracts for relevance and eligibility for full-text screening. Only reports that were obviously irrelevant were discarded. Using the Distiller-SR online software tool [13], we developed a full-text screening form for study selection. Disagreements concerning study inclusion were discussed until a consensus was reached.

### Data collection process

Two reviewers (AA and AG) performed the data extraction independently and in duplicate with the use of standard data extraction forms created in Distiller-SR. Predefined reasons for exclusion were documented for each of the excluded studies. Two reviewers assessed methodological quality of the included studies. Discrepancies were resolved by discussion, and if needed, a third reviewer (GN) was consulted. No individual patient data was used.

### Data items

For each included study, we extracted data on characteristics of study participants, study design, details of interventions including drug concentration, outcomes (count and rate data as applicable, with measures of dispersion), and methodological factors affecting risk of bias and other quality appraisal criteria.

### Methodological quality appraisal

We applied the GRADE quality appraisal criteria [10]. These include risk of bias [14], indirectness (in population, intervention or outcome) [15], imprecision [16], inconsistency (heterogeneity) [17], and publication bias [18]. Risk of bias in individual studies was assessed according to the criteria proposed by Higgins et al., including adequacy of sequence generation, allocation concealment, blinding of participants, outcome assessors, and study personnel, completeness of follow-up, and selective outcome reporting [19].

### Summary measures and synthesis of results

All analyses were performed using RevMan 4.3 for Macintosh. We used random effects models to compute rate ratios, but used fixed-effects models when only two studies were available for pooling [20]. We assessed heterogeneity using the I^2^ statistic.

### Additional analyses

Where possible, we performed (pre-specified) subgroup analyses according to citrate concentration, using cut-points defined by included studies (<5% or >30%).

## Results

### Study selection

**Figure 1:**
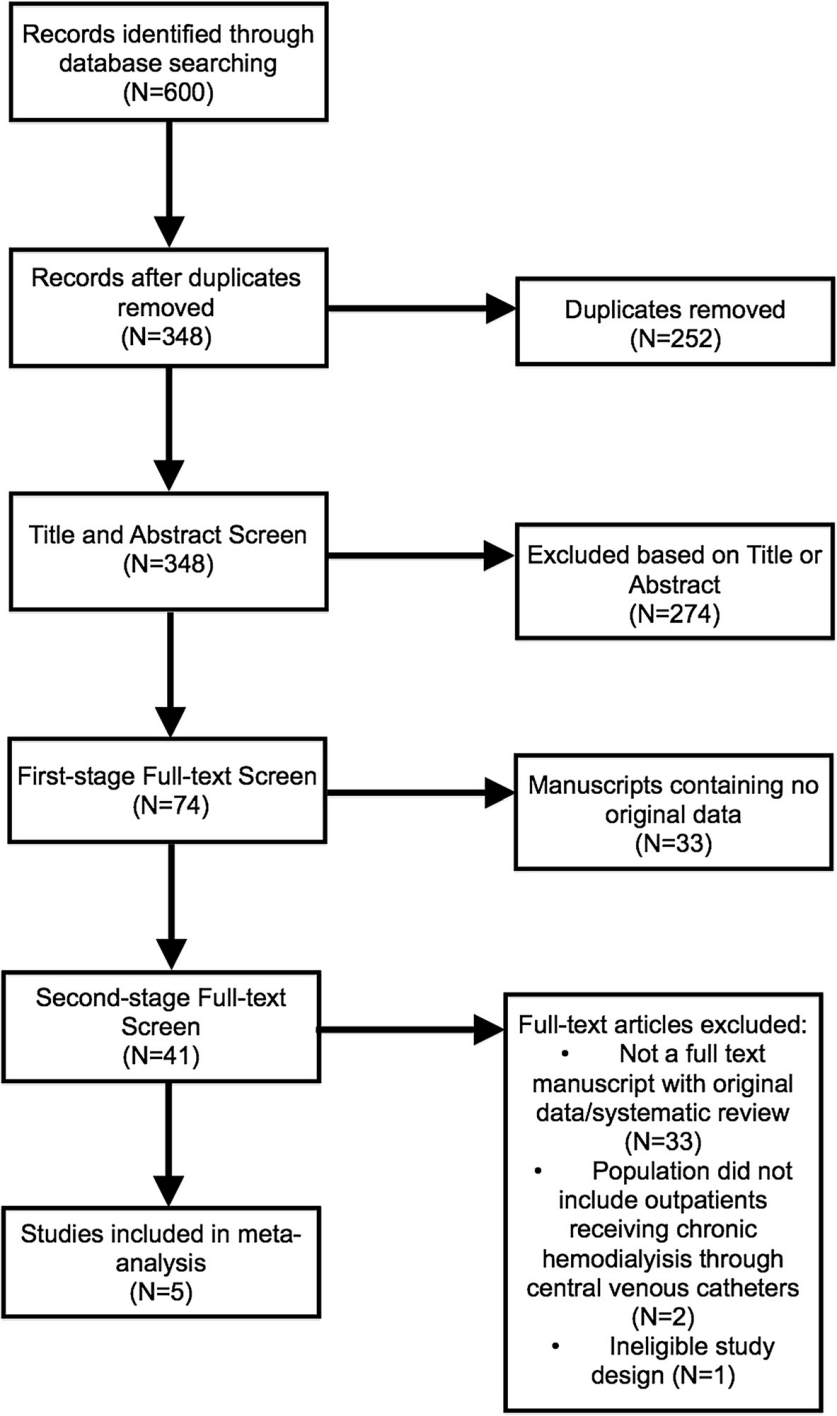
**Study inclusion flow diagram.**

We identified a total of 600 studies (Figure 1), including 111 in Medline, 31 in CINAHL, 268 in EMBASE, 31 in CCTR and 159 in Web of Science. We did not identify any relevant conference abstracts. We identified and removed duplicate records (N = 252). Forty-one studies proceeded to full-text screening. Reasons for exclusion were: no original data (including systematic reviews) (N = 33), study population did not consist of outpatients receiving chronic hemodialysis through central venous catheters (N = 2), and crossover design (N = 1). Five studies were therefore included in our review [21-25].

### Study characteristics

All 5 included studies were parallel-arm randomized trials (Table 1). Studies included between 19 and 291 participants (median = 61), followed for a total of 176.4 catheter-years, with study population mean age ranging between 62 to 75 in citrate treatment groups and between 61 to 71 in heparin treatment groups, and percentage of female patients ranging from 34 to 60 in citrate treatment groups and from 41 to 56 in heparin treatment groups. Studies were conducted in Canada, the Unites States, Europe, and Asia; two of the studies were multi-centered [23].

**Table 1:** **Characteristics of included studies**

**Source**	**Country (#Centres)**	**Study duration**	**N**	**Methods**	**Participants**	**Intervention**	**Control**	**Outcomes**	**Funding Source**
[22]	**Slovenia (1)**	**Not specifically stated.**	**30**	Method of randomization unclear.	ESRD patients with subclavian or jugular single lumen catheters inserted as temporary blood accesses for HD expected to be used for at least 7 days.	3 ml of 4% trisodium citrate	A mixture of 1 ml (5000 U/ml) of unfractionated heparin and 2 ml of saline.	Catheter removal.	Not specified.
[23]	**Belgium (1)**	**April 2000 - October 2000**	**19**	Method of randomization unclear.	Chronic hemodialysis patients with a single lumen central venous catheter as permanent vascular dialysis access.	5% trisodium citrate	5000 U/ml heparin solution	Local/in-situ thrombolysis (bolus events for inadequate blood flow).	Not specified.
[24]	**Canada (1)**	**December 2004 - June 2005**	**61**	Randomization based on patient surnames.	All patients receiving chronic HD three times a week, 4 h/session, at the in-center Hemodialysis Unit with cuffed catheters as primary vascular access were eligible for the trial.	Prefilled 5 ml syringe containing 4% citrate (MEDXL, Montreal, Quebec, Canada).	1 ml of 10,000 U/ml heparin diluted to 5000 U/ml with normal saline	Patient survival, bacteremia rates, catheter removal, and local/in-situ thrombolysis.	Not specified.
[25]	**United Kingdom (4)**	**6 mo. period**	**232**	Open-label. Randomization was achieved through single-random number allocation.	ESRD patients with IJ CVCs (Bio-Flex TC), on HD > 90 days.	46.7% trisodium citrate.	5% heparin	Patient survival, bacteremia rates, catheter-related hospitalization rates, catheter replacement/exchange rates, use of urokinase.	Not specified.
[26]	**Netherlands (10)**	**April 2001 - September 2002**	**291**	Randomization by computer-generated random number list.	Patients >18 years, not admitted to the intensive care ward, with chronic or acute renal failure that required hemodialysis through a CVC. Only patients with a newly inserted, well-positioned CVC expected to be in use for 1 wk.	30% trisodium citrate	Unfractionated sodium heparin 5000 U/ml	Patient survival, bacteremia rates, catheter-related infection hospitalization rates, catheter removal rates, and catheter treatment with urokinase.	Not specified.

Abbreviations: CVC – central venous catheter; HD – hemodialysis; IJ – internal jugular.

### Results of individual studies

Event rates for individual studies are in Table 2. Follow-up ranged between 1,703 and 19,008 catheter days for citrate treatment groups, and between 1,493 and 17,100 catheter days for heparin treatment groups across studies.

**Table 2:** **Rates of events in individual studies**

**Study**	**Citrate**			**Heparin**		
	***Events***	***Catheter days***	***Events/1000 catheter days***	***Events***	***Catheter days***	***Events/1000 catheter days***
**Mortality**						
Power	5	19008	0.26	5	17100	0.29
Weijmer	13	8181	1.59	18	8049	2.24
MacRae	4	2272	1.76	5	1818	2.75
**Bacteremia**						
Power	13	19008	0.68	12	17100	0.70
Weijmer	9	8181	1.10	33	8049	4.10
MacRae	5	2272	2.20	6	1818	3.30
**Access-related hospitalization**						
Power	17	19008	0.89	12	17100	0.70
Weijmer	6	8181	0.70	21	8049	2.70
**Catheter replacement**						
Buturovic	1	2272	1.96	1	1818	4.35
MacRae	8	2272	3.52	5	1818	2.75
Weijmer	27	8181	3.21	29	8049	3.56
**Thrombolysis**						
MacRae	13	2272	5.72	13	1818	7.15
Power	1	19008	0.06	1	17100	0.04
Weijmer	69	8181	8.28	63	8049	7.88
**Bleeding**						
MacRae	25	2272	11.00	37	1818	20.35
Weijmer	5	8181	0.60	16	8049	2.00

### Synthesis of results

**Figure 2:**
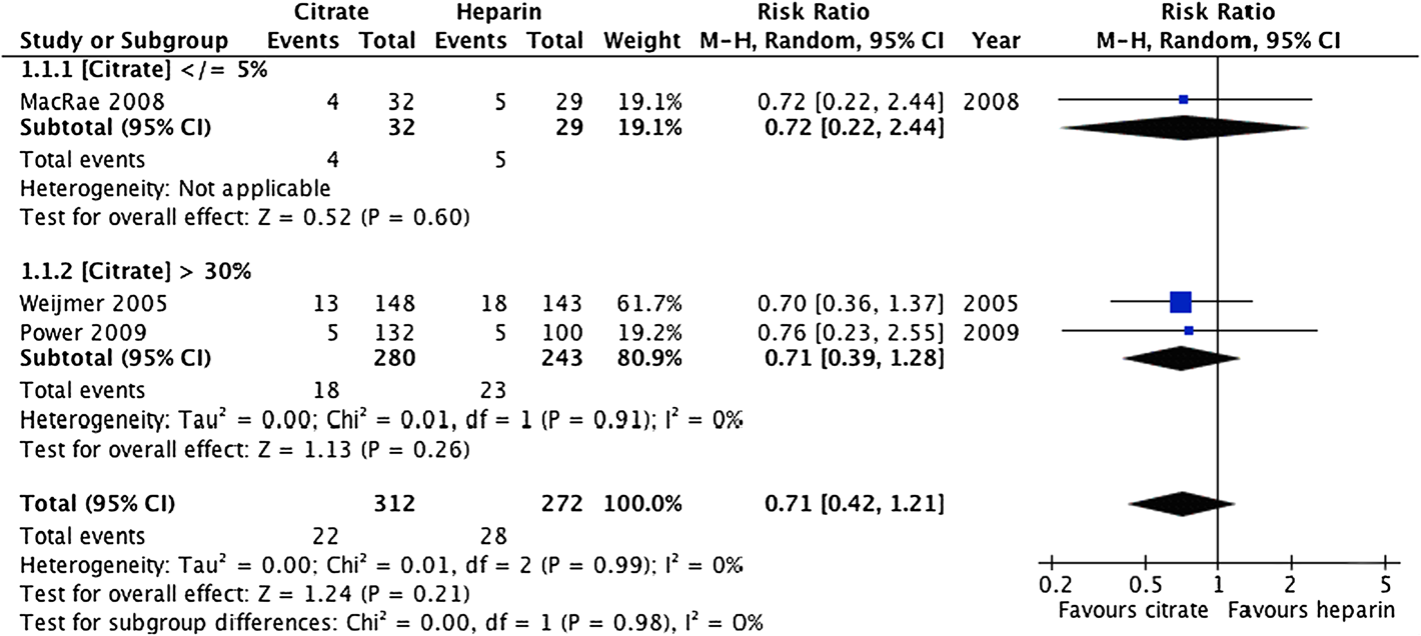
**Comparative risk of death (all-cause) with citrate vs. heparin locking solutions.**

**Figure 3:**
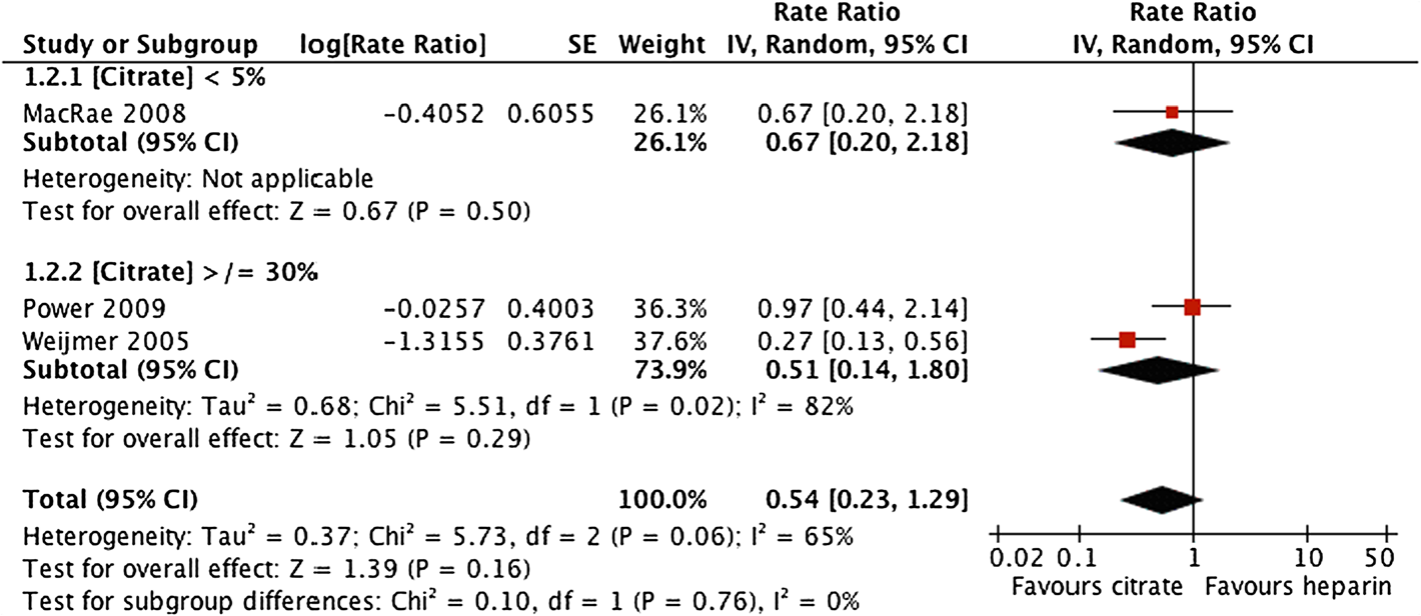
**Comparative risk of bacteremia with citrate vs. heparin locking solutions.**

**Figure 4:**
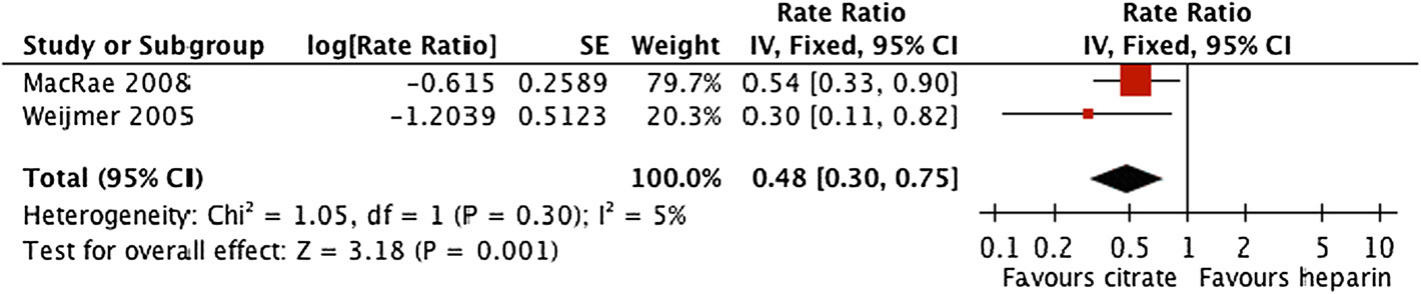
**Comparative risk of bleeding with citrate vs. heparin locking solutions.**

**Figure 5:**
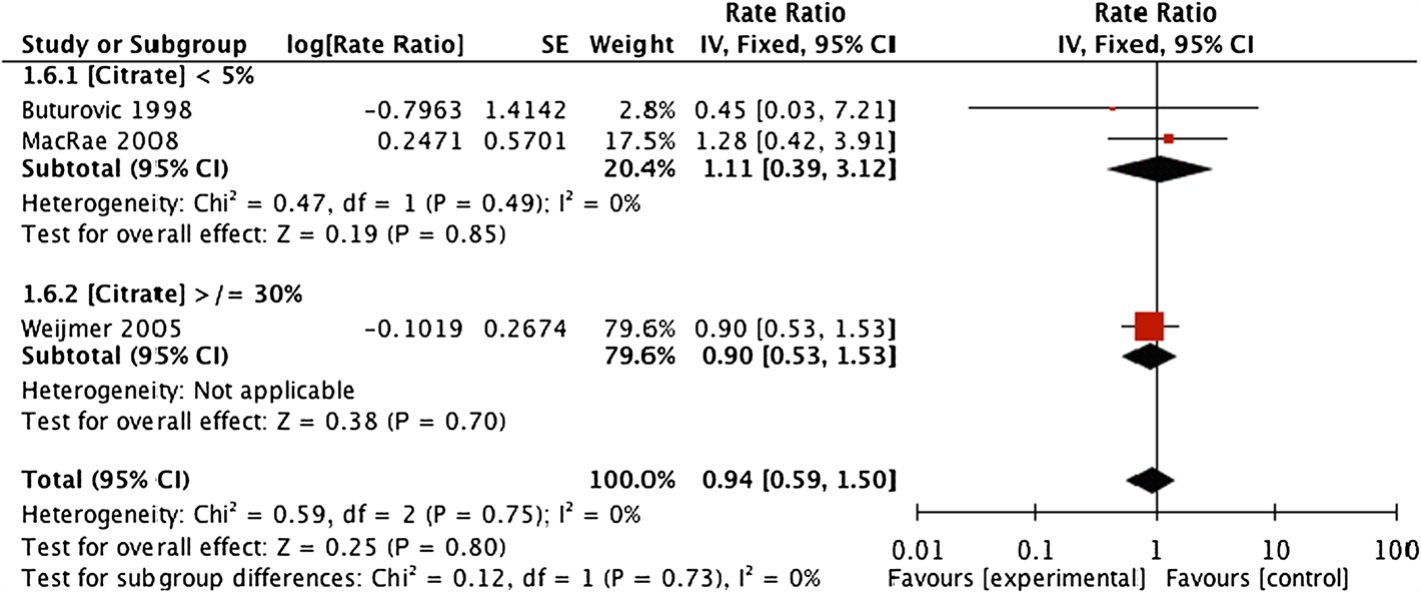
**Comparative risk of CVC replacement for impaired patency with citrate vs. heparin locking solutions.**

**Figure 6:**
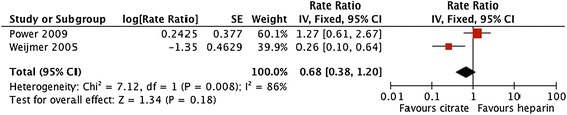
**Comparative risk of hospitalization (any cause) with citrate vs. heparin locking solutions.**

Forest plots and details of pooled estimates are in Figures 2, 3, 4, 5, 6 and Table 3. Three studies reported all-cause mortality, with a pooled relative risk of 0.71 (95% CI = 0.42-1.24; p = 0.21; I^2^ = 0%) favouring citrate over heparin. Three studies assessed bacteremic episodes; the rate ratio for this outcome was 0.54 (95% CI = 0.23-1.29; p = 0.16; I^2^ = 65%) favouring citrate over heparin. Bleeding was reported in two studies, with a rate ratio of 0.48 (95% CI = 0.3-0.75; p = 0.001;I^2^ = 5%), favouring citrate over heparin. Rates of catheter exchange/replacement and in-situ thrombolysis were not significantly different between groups in any of the pooled analyses. Finally, the pooled rate ratio for all-cause hospitalization was 0.68 (95% CI = 0.38-1.20; p = 0.18; I^2^ = 86%), favouring citrate over heparin.

**Table 3:** **GRADE evidence profile table: citrate vs. heparin locking solutions for hemodialysis catheters**

**Quality assessment**							**№ of patients**		**Effect**		**Quality**	**Importance**
**№ of studies**	**Study design**	**Risk of bias**	**Inconsistency**	**Indirectness**	**Imprecision**	**Other considerations**	**Citrate**	**heparin**	**Relative (95% CI)**	**Absolute (95% CI)**		
Survival (follow up: range 56,428 Total Catheter Days)												
3	randomised trials	not serious	not serious	not serious	very serious ^1^	none	22/312 (7.1%)	28/272 (10.3%)	**RR 0.71** (0.42 to 1.21)	30 fewer per 1000 (from 22 more to 60 fewer)	⨁⨁◯◯ LOW	CRITICAL
Bacteremia (follow up: range 56,428 Total Catheter Days)												
3	randomised trials	not serious	serious ^2^	not serious	serious ^3^	none	27/312 (8.7%)	51/272 (18.8%)	**Rate Ratio 0.54** (0.23 to 1.29)	0 fewer per 1000 (from 0 fewer to 0 fewer) ^9^	⨁⨁◯◯ LOW	CRITICAL
Thrombolysis (follow up: range 56,428 Total Catheter Days)												
3	randomised trials	not serious	serious ^4^	not serious	serious	none	235/172 (136.6%)	150/180 (83.3%)	**Rate Ratio 1.25** (0.76 to 2.05)	0 fewer per 1000 (from 0 fewer to 0 fewer)	⨁⨁◯◯ LOW	IMPORTANT
Bleeding (follow up: range 20,320 Total Catheter Days)												
2	randomised trials	not serious	not serious	not serious	serious ^5^	none	30/180 (16.7%)	53/172 (30.8%)	**Rate Ratio 0.48** (0.30 to 0.75)	0 fewer per 1000 (from 0 fewer to 0 fewer)	⨁⨁⨁◯ MODERATE	IMPORTANT
Hospitalization for any reason (follow up: range 52,338 Total Catheter Days)												
2	randomised trials	not serious	serious ^6^	not serious	serious ^7^	none	23/280 (8.2%)	33/243 (13.6%)	**Rate Ratio 0.68** (0.38 to 1.20)	0 fewer per 1000 (from 0 fewer to 0 fewer)	⨁⨁◯◯ LOW	IMPORTANT
Catheter replacement for patency (follow up: range 24,410 Total Catheter Days)												
3	randomised trials	not serious	not serious	not serious	serious ^8^	none	36/190 (18.9%)	35/182 (19.2%)	**Rate Ratio 0.94** (0.59 to 1.50	0 fewer per 1000 (from 0 fewer to 0 fewer)	⨁⨁⨁◯ MODERATE	IMPORTANT

MD – mean difference, RR – relative risk.

^1^Studies not powered for survival outcome; optimal information siz e criterion not met.

^2^I^2^=65% for pooled effect estimate; could not exclude heterogeneity due to study design, duration of follow-up, and citrate concentration.

^3^Overall event rates were low; studies were likely underpowered to detect statistically significant differences in bacteremia.

^4^I^2^=77% for pooled effect estimate, possibly due to unexplained heterogeneity in outcome definitions and study design.

^5^Vary small event rate and sample siz e; observed effect may be due to random error.

^6^I^2^=86% using fixed-effect model; unexplained heterogeneity exists.

^7^Confidence interval includes no effect.

^8^Optimal information siz e criterion not met.

^9^No explanation was provided.

### Methodological quality appraisal

Table 3 summarizes the quality appraisal for pooled estimates on an outcome-by-outcome basis. Risk of bias was low in all included studies. One study was open-label, but due to the nature of the reported outcomes, unblinding was unlikely to result in a significant risk of bias [23]. A small overall number of included studies precluded any meaningful analysis of publication bias by funnel plots. Despite a low risk of bias within included studies, the overall quality of evidence was low for both critical and important outcomes, with inconsistency (heterogeneity) and imprecision (due to small sample sizes) affecting the quality of the majority of estimates.

### Additional analyses

We created subgroups according to citrate concentration (<5% vs. >30%). However, given the small overall number of studies, no significant subgroup effects were noted.

## Discussion

### Summary of evidence

Compared with heparin, citrate catheter locking solutions were associated with significantly fewer bleeding episodes. Rates of death and bacteremia tended to be lower with citrate, but pooled effect estimates were not statistically significant. No significant differences in catheter exchange/replacement, thrombolysis, or all-cause hospitalization were evident between groups in any of the pooled analyses.

Our findings are consistent with those of a similar and recently published meta-analysis by Zhao et al., which also found significantly reduced bleeding rates, but no difference in survival, hospitalization, or catheter patency with citrate alone as compared with heparin [26]. The review by Zhao et al. also included studies in which citrate was combined with various antimicrobial agents, including gentamicin, taurolidine, methylene blue, and methylparaben, yielding a pooled rate ratio of 0.39 (95% CI = 0.27 to 0.56; I^2^ = 27%) for catheter-related bloodstream infection, favouring citrate-containing locking solutions over heparin. Their findings are corroborated by two other similar reviews [27,28]. Given the small overall number of studies comparing the effects of citrate alone versus heparin (N = 3) on bacteremia rates, neither our study, nor that of Zhao et al. could exclude a beneficial effect on this outcome. Whether the protective effect of citrate on blood-stream infection rates noted by Zhao et al. is a result of greater statistical power due to the inclusion of more studies, or attributable to the secondary antimicrobial agents included in these study interventions is unclear. Larger studies would be needed to confirm whether or not citrate alone confers a benefit similar to that of citrate-based locking solutions containing additional antimicrobial agents.

Plausible biological mechanisms exist by which citrate alone might reduce systemic infections when instilled in catheter ports. In vitro studies have demonstrated bactericidal and sporocidal activity with both high (23%) and lower (4%) citrate concentrations, and prevention of biofilm formation, with no evidence of citrate resistance [29,30]. This is in contrast to heparin, which may in fact promote biofilm formation and increase infection risk [31,32]. Citrate’s antibacterial activity appears to be related to the chelation of calcium and magnesium ions, leading to the degradation of bacterial cell membranes, thus reducing bacterial cellular integrity [33]. Given its avidity for divalent cations, inadvertent systemic administration of large volumes of citrate locking solutions can result in calcium complexation and hypocalcemia. One report described a fatal outcome associated with high concentration citrate (46.7%) administration; this ultimately led to FDA and Health Canada bans on highly concentrated citrate locks in the US and Canada [34]. Our review did not identify any evidence of harm due to inadvertent systemic administration with the citrate concentrations used in included studies, and in current routine use [23].

We observed a significantly lower rate of bleeding with citrate as compared with unfractionated heparin (Figure 4). Although the magnitude of effect was similar in the two studies included in this pooled estimate, the event rates were vastly different (~10-fold difference in control group event rate; Table 2), suggesting differences in study populations, co-interventions, and outcome definitions. Assuming that bleeding risk attributable to locking solutions is due to occasional inadvertent systemic administration, it seems reasonable to conclude that the anticoagulant effect of citrate in these two studies (4% and 30%) was lower *in vivo* than were similar volumes of unfractionated heparin at 5000 IU/ml.

### Limitations

We included only randomized trials focusing on patient-important outcomes, and performed a rigorous methodological quality appraisal based on the GRADE approach. Nevertheless, some additional factors limit our overall confidence in the observed pooled estimates. A small overall number of included studies, small sample sizes within studies, and low event rates limit the precision of most of the pooled analyses. We also found some heterogeneity in the pooled estimate for bacteremia, but a small overall number of studies precluded any meaningful subgroup analyses. We also could not exclude publication bias.

### Implications for practice

Compared with unfractionated heparin, citrate catheter locking solutions appear to reduce bleeding, and may reduce bacteremia in patients undergoing hemodialysis with a CVC. However, it is unclear from the current published evidence whether citrate alone has the same protective effect against systemic infection as it does when combined with other antimicrobial agents, as observed in the review by Zhao et al. [26]. Although the overall quality of evidence supporting its use is low, both The American Society of Diagnostic and Interventional Nephrology (ASDIN) and European Renal Best Practice (ERBP) consider 4% citrate and heparin acceptable alternatives for locking CVCs [35,36]. Given the availability of 4% citrate in prefilled syringes, and lower unit costs when compared with unfractionated heparin, citrate may be the preferred choice, especially if differences in cost increase over time.

### Implications for research

Studies comparing citrate with unfractionated heparin-based catheter locking solutions have generally been small and not adequately powered to provide conclusive evidence of safety and efficacy; hence, larger comparative trials are desirable. Moreover, with the increasingly favourable safety profile and falling costs of low molecular weight heparins, studies comparing these agents with citrate would be useful in informing practice as well. Although not in widespread use, recombinant tissue plasminogen activator (rt-PA) has been used weekly for locking dialysis catheters, with cost effectiveness comparable to that of heparin (costs of rt-PA offset by reduced hospitalization for bacteremia) [37,38]. Future studies comparing the effectiveness of rt-PA with that of citrate might also be of interest. Finally, we, and others have noted significant variability in outcome definitions in hemodialysis vascular access studies [39]. This represents a potential source of heterogeneity and renders the overall body of evidence less amenable to meaningful quantitative synthesis. Standardization of outcome reporting in vascular access research is needed, and published outcome definitions now make this possible [40].

## Additional file

Additional file 1:
**Web Appendix - Search Strategy.** Search Strategy Example - MEDLINE.
