# Supplementary material for: Benefits and harms of citrate locking solutions for hemodialysis catheters: a systematic review and meta-analysis
Source: Can J Kidney Health Dis. 2015 Apr 2;2:13. doi: 10.1186/s40697-015-0040-2 (PMC4413999; doi:10.1186/s40697-015-0040-2)
Supplement: Additional file 1: — Web Appendix - Search Strategy. Search Strategy Example - MEDLINE. [file 40697_2015_40_MOESM1_ESM.docx]

Web Appendix - Search Strategy

Database: Ovid MEDLINE(R) Daily Update <June 27, 2013>, Ovid MEDLINE(R) In-Process & Other Non-Indexed Citations and Ovid MEDLINE(R) <1946 to Present>

Search Strategy:

--------------------------------------------------------------------------------

1 exp Renal Dialysis/ or exp renal replacement therapy/

2 (hemodialysis or haemodialysis).mp.

3 (hemodia* or haemodia* or hemofilt* or haemofilt* or diafilt*).mp.

4 dialysis.mp.

5 exp Hemofiltration/

6 or/1-5

7 exp Citrates/

8 (citrate* or citric acid).mp.

9 2 hydroxy 1,2,3 propanetricarboxylic acid.mp.

10 2 hydroxytricarballylic acid.mp.

11 acidum citricum.mp.

12 hemiacidrin.mp.

13 renacid.mp.

14 renacidin.mp.

15 renacidine.mp.

16 or/7-15

17 exp Catheters, Indwelling/ or exp Catheters/ or exp Central Venous Catheters/

18 exp Catheterization/

19 catheter*.mp.

20 or/17-19

21 lock*.mp.

22 20 or 21

23 6 and 16 and 22

***************************
